# Capital structure and financial performance of China’s energy industry: What can we infer from COVID-19?

**DOI:** 10.1371/journal.pone.0300936

**Published:** 2024-06-06

**Authors:** Ahmed Samour, Abdullah AlGhazali, Mihaela Gadoiu, Mariana Banuta

**Affiliations:** 1 Department of Finance and Economics, Dhofar University, Salalah, Oman; 2 Finance Department, Dhofar University, Salalah, Oman; 3 Department of Finance, Accounting and Economics, National University of Science and Technology Politehnica Bucharest, Pitesti University Center, Pitesti, Romania; Government College University Faisalabad, PAKISTAN

## Abstract

The study aims to uncover the impact of COVID-19 and capital structure on the financial performance of 1787 renewable and nonrenewable energy firms in China from 2010 to 2022. Using the fixed effect approach, our study found that financial leverage negatively affected the return on assets and equity ratios for both renewable and nonrenewable energy. On the other hand, the study shows that COVID-19 adversely affected the financial performances of non-renewable energy firms. Conversely, COVID-19 positively affected the financial performances of renewable energy firms. The conclusions drawn by the present study are helpful for the policymakers in making corresponding financial decisions. The study suggests that policymakers must adopt profitable capital structure strategies for firms and shareholders in this context. Finally, policymakers must design more policies to overcome the adverse influence of the COVID-19 pandemic crisis and avoid any future unforeseeable pandemics.

## 1. Introduction

The capital structure implies how the firm finances its assets through its mix of debt and equity, which is recognized as one of the main significant decisions made by the management since it may affect the firm performance [1]. The recent literature demonstrated that a firm’s profitability is influenced by capital structure decisions [e.g., 2, 3]. However, the evidence on the relationship may be positive or negative. This work aims to examine the importance of capital structure on the energy firm’s performance in the case of China. In this context, the present study seeks to answer two essential questions. First, what are the effects of the capital structure on financial performance in the leading renewable and nonrenewable energy firms in China?. Second, what are the effects of COVID-19 on financial performance in the renewable and nonrenewable energy firms?. Although there are several empirical works on the capital structure-financial performance link in the literature [e.g., 4–8]. Few studies assessed this nexus in the energy sector [2, 9–11]. In this context, [8] suggested that financial leverage, which is used as a proxy to measure capital structure, negatively affected ROE and ROA in Malaysian oil and gas companies. [12] assessed how capital structure in US energy firms can affect performance. The authors affirmed that financial leverage has an adverse correlation with the financial performance from 2009 to 2019 period. [9] demonstrated that financial leverage negatively correlated with the financial performance of energy firms in the USA. On the other hand, [10] showed that an increase in the debt ratio has no significant impact on the financial performance of Indian firms. Recent studies considered the effect of the COVID-19 pandemic on firms performance and values [11, 13–16]. For example, [11] found that COVID-19 adversely affected the energy sector’s performance. [14] demonstrated that the COVID-19 pandemic adversely influenced the performance of energy firms in the case of China. According to the gaps observed in the existing empirical studies, the present work aims to contribute the finance and accounting literature in the following ways. First, few studies evaluated the capital structure-financial performance linkage in the energy sector. Most of the empirical papers ignored the impact of the capital structure-financial performance in the China’s energy sector. Besides, most empirical studies ignored the impact of capital structure on financial performance of energy industry in response to COVID-19. In this context, the study aims to present fresh insight to the frontiers of the empirical literature on the impact of capital structure, liquidity on financial performance in response to COVID-19 in by considering both renewable and nonrenewable energy firms in China. To the best of authors’ knowledge, this study is the first to investigate this relation in case of China. Several reasons to focus on the energy industry especially in case of China. The global trend of increasing energy demand has led to significant investment development in the energy sectors in China [1]. In this context, this industry has powerful influence on the country economic and financial development. Hence, energy sector in the country is considered one of the most vastly growing sectors in terms of its contribution to economic development. This development has a powerful influence on the firm structure due to mergers to exploit scale and scope economies. One of the ways to mitigate the costs of energy investment is the correct decisions regarding the financing of the firm. Likewise, due to its significant impact on the country’s environment sustainability, it is substantial to understand the nexus among capital structure and firm performance in both renewable and nonrenewable energy firms. Understanding this relationship is important to reinforce the funds for green energy firms. Eventually, this will promote the environment sustainability in the country. Therefore, the percent study aims to offer valuable policy implication to Chinese policy makers to sustain the market and the environment.

COVID-19 infections have rocked businesses worldwide, assessing the resilience of several firms to the pandemic [17]. To overcome the COVID-19 crisis, governments started implementing social distancing measures such as suspension of study and work [18]. This situation negatively affected the level of production and manufacturing. Therefore, global energy demand significantly declined in the first months of this pandemic. In this context, the drop in the demand of oil and gas at the beginning of 2020 undoubtedly affected the profitability of global energy firms.

China, as the world’s second largest economy, has had tremendous growth in gross domestic product (GDP) in the last decades [19]. The significant improvement in financial and economic development has been accompanied by a significant increase in energy investment and consumption [20]. In this context, China generates the largest amount of renewable energy sources [21]. The local government designed several policies to promote the renewable energy sector, such as formulating benchmark feed-in tariffs for green energy sources [22]. The government’s in China subsidy to the renewable energy industry increased by 100% from 2008 to 2022 [23, 24].

This paper is organized as follows: section 2 presents the theoretical framework and the literature review. Section 3 shows data and methodology. The empirical findings are stated in section 4 and section 5 concludes the paper.

## 2. Theoretical framework and literature review

### 2.1 Theoretical framework

The literature on capital structure is very comprehensive, Modigliani and Merton Miller theorem as proposed by [25], who are considered the foundation for capital structure theories. According to [25] the capital structure has no significant influence on financial performance. This theory assumes that there are no transaction costs or taxes. In addition, the theory also assumes that the rates of lending and borrowing rates are equal, and information is symmetric. Besides, all market investors have the same expected rate of return and risk. These assumptions received significant criticisms, such as the absence of transaction costs and tax-perfect capital markets. However, [26] assessed the capital structure determined by taking into account tax advantages. In this line, the tax is considered as a primary determinant of capital structure, which assumes that firms finance their assets from debt to make use of tax advantages and to maximize the firms’ profitability. [27] proposed agency theory, which assumes conflicting interests exist among shareholders and managers. This theory argues that firms with a high level of debt is under pressure to improve their liquidity position to pay interest on time, which in turn leads to an improvement the firm performance [27].

[28] proposed the trade-off theory, which depicts that the firms choose the optimal capital structure for their firms where it trades off among the costs of the debts and its advantages. Based on this theory, the firms will compare the debt and equity sources. The capital structure will differ for every firm depending on the firm characteristics. [29] proposed the Pecking Order Theory, which is formulated on the assumption that any firm follows a specific order to finance its projects and investments. However, in this theory, internal financing sources such as retained earnings are preferred over external financing, followed by debt financing and choosing equity financing as a last resort [29]. Therefore, external financing raises perception of the market, thus growing a firm’s value. [30] proposed market timing theory and suggested that stock price volatility has a powerful influence on the firm capital structure decisions. However, [30] states that there are no optimal capital structure decisions. Besides, he argues that market conditions significantly affect capital structure decisions.

### 2.2 literature review

Several empirical works have assessed the capital structure-financial performance link. Some of the empirical studies illustrated a positive link among capital structure and firms’ performance. For example, [3] assessed the influence of capital structure on the profitability on the Indian firms over the 2008 to 2012 period. The author found that an increase in debt level will positively affect performance. [4] showed a significant positive association between Australian firms’ return on equity (ROE) and leverage. [31] evaluated the effect of financial leverage on the performance of Indian microfinance firms. The study demonstrated a positive and significant linkage among financial leverage and performance. Using data from Argentina, Mexico, Colombia, Brazil, Peru, and Chile from 1997 to 2013, [5] demonstrated that capital structure is a significant driver of the firm performance. [2] found that leverage is positively associated with ROE in firms listed in the Indian stock market. Similarly, the study of [32] reveals a significant positive impact of leverage on ROE in firms listed China’s stock market. However, other empirical studies illustrated an adverse interaction among debt and financial performance. [6] used a data set from 285 non-financial firms and empirically showed that financial leverage negatively affects the financial performance of Pakistani firms. [7] used the data set from the 2010 to 2014 period and demonstrated that an increase in financial leverage negatively affected the Nigerian firm’s performance. In the energy sector, [8] examined the interconnection amid capital structure and the performance of Malaysian oil and gas firms. The author showed that financial leverage negatively affected ROE and ROA in the tested oil and gas firms in Malaysia from 2003 to 2013. [12] assessed the effect of financial leverage on the USA’s energy firms. The study affirmed that financial leverage is adversely correlated with financial performance over the period from 2009 to 2019. Similarly, the findings of [9] study affirmed that financial leverage is negatively associated with the financial performance of the energy firms in the USA. On the other hand, [10] used the sample of 140 energy firms in India and showed that an increase in the ratio of debt has no significant influence on the performance.

Recently, [33] used data of 200 Turkish firms from 2009 to 2019. Using the Pooled Ordinary Least approach, the authors found that an increase in financial leverage negatively affects financial performance. [34] used a global sample of 646 firms from 2010–2018. The authors used the panel OLS approach and suggested that financial leverage adversely influences financial sustainability in the tested firms. [35] explored the effect of financial leverage on firms’ performance in Germany from 2002–2018. The authors found that financial leverage decreases the financial performance of the tested firms over the examined period.

The COVID-19 pandemic has triggered severe economic development since January 2020. However, the impact of COVID-19 on the performance of energy firms has attracted some of the author’s attention. [11] highlights the impact of COVID-19 on energy manufacturing levels. The author demonstrated that COVID-19 negatively affected the energy sector’s performance. Likewise, [36] showed that COVID-19 harms firms’ performance in China. [37] employed the quarterly data of 107 economies and showed firms’ performance from 2020Q1 to 2020Q3 was negatively affected by COVID-19. In the energy sector, [14] showed that the COVID-19 pandemic adversely influenced the performance of energy firms. The authors showed that the energy firms faced an increase in debt levels, which in turn negatively affected their firm performance in China. [15] affirmed that COVID-19 adversely affects the equity crash risk of energy firms in China. [16] also found that COVID-19 negatively affected the performance of Greek energy firms. In light of the above empirical, this study aims to contribute to the finance and accounting literature in the following two ways: First, several empirical works on the capital structure-financial performance link in the empirical papers [e.g., 4–8]. Few empirical works evaluated this relationship in energy firms. However, most current studies ignored the impact of capital structure on financial performance by considering the COVID-19 pandemic. Therefore, this paper fills the finance and accounting literature gap by evaluating the capital structure on the performance of energy firms in response to the COVID-19 crisis by considering both renewable and nonrenewable energy firms in China.

## 3. Data and methodology

### 3.1 Data

This work examines the impact of capital structure, liquidity, and COVID-19 on the financial performance of China’s leading renewable and nonrenewable energy firms. We obtained our data from the Refinitiv Eikon database over the period from 2010 to 2022. The sample size consists of 1787 energy firms. We further split the sample into nonrenewable energy firms (N = 1282) and renewable energy firms (N = 505). The dimensionality issue of the employed model setting and data variability can explain why we selected these energy firms. We applied the ratios of the return on equity (ROE) and return on assets (ROA) as proxies to capture the financial performance of the energy firms [38]. ROE and ROA are market-based measures of financial performance. These ratios are measured as net income divided by total equity and total assets. Return on equity is measured as:

$$ROA=\frac{Net\;income}{Total\;assets}$$

$$ROE=\frac{Net\;income}{Book\;value\;of\;equity}$$

A firm’s leverage (LEV) is calculated using a debt-to-assets ratio. We used the firm’s leverage as a proxy to measure the capital structure [39]. Liquidity (LIQ) is measured using the current ratio (current assets/current liability).

Furthermore, we include Size, Tangibility (TAN) and Age as additional control variables that have been documented to influence energy firms (e.g. [38, 40]). We measure tangibility as fixed assets scaled by total assets, and age is the logarithm of firm age since inception. We also control the COVID-19 pandemic as a dummy variable equals to 1 for years 2020 and 2021, and 0 otherwise. All continuous variables are winsorized at 1 and 99 levels to eliminate the impact of the outliner on our estimations. Table 1 reports the definitions of variables used in this study.

**Table 1:** The definitions of the selected variables.

Name	Definitions
*ROE*	ROE captured as “net income” scaled by book value of equity
*ROA*	ROE captured as “net income” divided by total assets
*LEV*	Leverage captured dividing the total debt to book value of total assets
*LIQ*	Liquidity: ‘current assets’ scaled by current liabilities
*Size*	Total assets in logarithm
*TAN*	Tangibility: fixed assets scaled by total assets
*Age*	Firm age: captured as the logarithm of firm age since inception
*COVID*	Dummy variable equals to 1 for years 2020 and 2021, and 0 otherwise

### 3.2 Methodology

This research examines the effects of leverage, liquidity, and other control variables on energy firms’ performance in China. We follow the recent studies of [7, 41] estimate a linear linkage among capital structure, liquidity and firm performance as follows:

$${Performance}_{i,t}=\;\beta_{0}+\;\beta_{1}\;{LEV}_{i,t}+\;\beta_{2}\;{LIQ}_{i,t}+\;\beta_{3}\;{Size}_{i,t}+\;\beta_{4}\;{TAN}_{i,t}+\;\beta_{5}\;{Age}_{i,t}+\varepsilon_{i,t}$$ (1)

$${Performance}_{i,t}=\;\beta_{0}+\;\beta_{1}\;{LEV}_{i,t}+\;\beta_{2}\;{\;{LEV}_{i,t}*COVID+\;\beta_{3}LIQ}_{i,t}+\;\beta_{4}\;{Size}_{i,t}+\;\beta_{5}\;{TAN}_{i,t}+\;\beta_{6}\;{Age}_{i,t}+\;\beta_{7}COVID+\varepsilon_{i,t}$$ (2)

$${Performance}_{i,t}=\;\beta_{0}+\;\beta_{1}\;{LEV}_{i,t}+\;\beta_{2}\;{LIQ}_{i,t}+{\;\beta_{3}\;LIQ}_{i,t}*COVID+\;\beta_{4}\;{Size}_{i,t}+\;\beta_{5}\;{TAN}_{i,t}+\;\beta_{6}\;{Age}_{i,t}+\;\beta_{7}COVID+\varepsilon_{i,t}$$ (3)

where performance is the return on equity (ROE) and the return on assets (ROA). LEV is the firm leverage, LIQ is the liquidity ratio, Size is the logarithm of total assets, TAN is the assets tangibility, age is the age of the firm, and COVID represents the dummy variable that takes the value of 1 for the pandemic years (2020 and 2021), and 0 otherwise [42].

Before estimating the link among the tested variables, we applied Pearson correlation analysis test to check the multicollinearity among data. Likewise, the endogeneity issues should be considered when assessing the linkage among the tested variables [43]. In this context, the fixed effect model is usually used to mitigate the impact of possible unobserved heterogeneity and avoid the endogeneity and correlation issues in the employed variables and models. This model can be used to analyze panel data because it allows us to control for unobservable variables [44]. This approach calculates the true effect size via the weighted average mean of effect sizes reported in the work.

## 4. The empirical findings

Our study aims to uncover the effect of capital structure, liquidity, and COVID-19 on the financial performance of the whole sample (panel A), non-renewable (panel B) and renewable (panel C) energy Chinese firms. Table 2 displays the descriptive statistics. Panel A of Table 2 shows that the mean return on equity (ROE) is 1% with a median of 7%. The ROA is about 3% with minimum of -191% and a maximum of 80%. The capital structure (Leverage) has a mean of 57%. The liquidity ratio of energy firms in China has a mean of 131% with a minimum 6%. Our sample contains the different size of energy firms, with a minimum of 17.6 and a maximum of 28.6. Tangibility has a mean of 55%. The age of the firms ranges from 4 to 38 years with a mean of 15 years.

**Table 2:** Descriptive statistics.

	Mean	Median	Min	Max	Std. Dev.
Panel A: Whole Sample (N = 1787)					
ROE	0.01	0.07	-24.71	37.39	1.45
ROA	0.03	0.03	-1.91	0.80	0.10
LEV	0.57	0.57	0.20	1.00	0.17
LIQ	1.31	1.12	0.06	11.22	0.91
Size	22.91	22.87	17.69	28.64	1.72
TAN	0.55	0.55	0.20	0.96	0.19
Age	14.99	15.00	1.00	38.00	6.73
Panel B: Non-Renewable Energy (N = 1282)					
ROE	0.03	0.07	-24.71	37.39	1.53
ROA	0.03	0.03	-1.91	0.80	0.10
LEV	0.56	0.57	0.20	1.00	0.18
LIQ	1.28	1.08	0.06	11.22	0.90
Size	23.02	23.06	18.32	28.64	1.82
TAN	0.58	0.61	0.20	0.96	0.20
Age	15.93	16.00	1.00	38.00	6.60
Panel C: Renewable Energy (N = 505)					
ROE	-0.03	0.08	-16.48	1.22	1.21
ROA	0.03	0.03	-0.40	0.44	0.08
LEV	0.58	0.58	0.20	0.99	0.16
LIQ	1.38	1.18	0.21	9.75	0.91
Size	22.62	22.49	17.69	25.70	1.38
TAN	0.48	0.47	0.20	0.92	0.16
Age	12.97	13.00	1.00	34.00	6.57

This table shows several characteristics of the selected energy firms. It shows the mean, median, max, minimum and ‘standard-deviation’ of the examined variables for whole sample (N = 1787) in Panel A; Non-Renewable Energy firms (N = 1282) in Panel B and Renewable Energy firms (N = 505) in Panel C. All variables are defined in Table 1.

Table 2 also displays the descriptive statistics for each group: Non-renewable energy (Panel B) and renewable energy (Panel C). Non-renewable firms are more profitable, larger, and invest more on fixed assets than renewable energy firms. The leverage and liquidity of both types of energy firms are very similar. These characteristics affirm the importance of the renewable energy sector in China.

Before estimating the link among the tested variables, the study used the correlations test to assess correlations among explored variables, as stated in Table 3. The correlation matrix shows that financial performance (ROE and ROA) is negatively correlated with tangibility (TAN), leverage (LEV), and age and financial performance measures (ROA and ROE). Furthermore, Table 3 shows that liquidity (LIQ) and the firm’s size (Size) are positively associated with profitability measures. The findings from Table 3 show that all variables’ correlations are lower than the 0.80, indicating no issue with multicollinearity.

**Table 3:** Correlation matrix.

* *	ROE	ROA	Leverage	Liquidity	Size	Tangibility	Age
ROE	1						
ROA	0.8264*	1					
LEV	-0.1749*	-0.2949*	1				
LIQ	0.0562*	0.1526*	-0.6535*	1			
Size	0.0890*	0.0387	0.3585*	-0.3329*	1		
TAN	-0.0341	-0.0502*	0.2689*	-0.4154*	0.4848*	1	
Age	-0.0543*	-0.1063*	0.0680*	-0.0734*	0.2733*	0.1743*	1

This table shows the pairwise correlations amongst the selected variables for the firm-specific factors. The figures in **bold** imply that the coefficient is significant at the 1 percent significance level.

As mentioned above, this study aims to examine the impact of energy firms’ characteristics on their financial performance (ROA and ROE) by employing a fixed effect model. We further examine the validity of fixed effect models in our study by conducting different tests. First, we employ huasman test to choose between random or fixed effect models. The estimated output in Panel A of Table 4 reveals that a rejection of the null hypothesis of no fixed-effects at 5% level of significance. Second, a test for heteroscedasticity in the fixed effect model is carried out using Modified Wald test for group wise heteroscedasticity where the null hypothesis is homoskedasticity. The result implies that we reject the null hypothesis (Prob>chi2 = 0.000). Therefore, we estimate fixed effect model with robust standard error in all models.

**Table 4:** Diagnostics.

**Panel A**. Huasman test		
	chi2 (8)	17.96
	Prob > chi2	0.0030
**Panel B**. Heteroscedasticity test		
	chi2 (62)	2.6e+05
	Prob > chi2	0.000

The estimated outputs of Eq (1) is presented in Table 5. The independent variable in models 1 to 3 (models 4 to 6) is ROE (ROA). Models 1 and 4 display the findings for the whole sample, models 2 and 5 report the findings of non-renewable energy, and models 3 and 6 reveal the results of the renewable energy subsample.

**Table 5:** The determinants of performance.

Dependent Variables	ROE			ROA		
	Model 1	Model 2	Model 3	Model 4	Model 5	Model 6
Constant	-0.191	0.388	-1.087	0.0193	0.267**	-0.402**
	(-0.52)	(0.86)	(-1.46)	(0.18)	(2.10)	(-2.03)
LEV	-0.488***	-0.505***	-0.356*	-0.149***	-0.151***	-0.118***
	(-5.96)	(-5.58)	(-1.78)	(-7.38)	(-7.43)	(-2.84)
LIQ	-0.0184***	-0.0171***	-0.0206**	-0.00416***	-0.00413**	-0.00403**
	(-4.56)	(-3.78)	(-2.22)	(-3.24)	(-2.57)	(-2.47)
Size	0.0329*	0.00506	0.0762*	0.00770	-0.00415	0.0284***
	(1.85)	(0.24)	(1.86)	(1.49)	(-0.72)	(2.80)
TAN	-0.277***	-0.185**	-0.512***	-0.0780***	-0.0467*	-0.154***
	(-3.33)	(-2.27)	(-2.77)	(-3.05)	(-1.91)	(-2.83)
Age	-0.00430	-0.00379	-0.00702	-0.00213***	-0.00176**	-0.00424*
	(-1.57)	(-1.42)	(-0.68)	(-2.97)	(-2.47)	(-1.72)
Observations	1787	1282	505	1787	1282	505
Group	163	112	51	163	112	51
R-Square	0.0827	0.0951	0.0954	0.122	0.143	0.155
Adj R-Square	0.0801	0.0916	0.0863	0.120	0.139	0.146
P-value	0.00	0.00	0.016	0.00	0.00	0.00

This table reports fixed effect estimation of the effects of capital structure and liquidity on energy firm’s performance. The independent variable is ROE and ROA in models 1 to 3 and models 4 to 6, respectively. Models 1 and 4 report the estimated outputs regarding the determinants of firms’ performance for the whole sample. Models 2 and 5 report the estimated outputs regarding the determinants of firms’ performance for the whole sample excluding renewable energy firms. Models 3 and 6 report the estimated outputs regarding the determinants of firms’ performance for the renewable energy firms. All other variables are presented in Table 1. The numbers in ‘parentheses’ indicates to the (*t*−statistics) values. *,**,*** represent the significance levels at (10, 5, and 1 percent)respectively.

The results in Table 5 show that financial leverage (LEV) is adversely correlated with the financial performance of renewable and non-renewable energy firms. The findings show that for the whole sample (models 1 and 4) a 1% significance level increase in financial leverage will decrease ROE and ROA by 0.48% and 0.149%, respectively. Besides, the findings show that a 1% significance level increase in financial leverage will reduce ROE by 0.50% and ROA by 0.151% for non-renewable energy firms, as shown in models 2 and 5. Additionally, considering the estimated results for the renewable energy firms subsample (models 3 and 6), the correlation coefficient showed the strongest inverse linkage among financial leverage and profitability measures. The findings show that a 1% significance level increase in financial leverage will reduce ROE and ROA by 0.356 and 0.118, respectively.

Despite the coefficient of financial leverage- financial performance linkage in non-renewable energy higher than renewable energy firms, it is clear that the findings of this study provide strong evidence that financial leverage has a strong negative relation with financial performance for both renewable and nonrenewable energy firms. These findings affirm that capital structure impacted the financial performance of the energy firms in the case of China. An interpretation of this is that the high debt ratio of the renewable and nonrenewable energy firms could give markets the negative signal that these firms could not mobilize funds from the stockholders. However, the study’s findings align with the pecking order theory, which assumes a negative relationship between debt ratio and profitability because firms use internal financing as the first financing source before seeking external financing.

The empirical findings with the two dependent variables described above demonstrate that liquidity affect negatively the financial performance (ROA and ROE) with a coefficient value of -0.0184% and -0.00416%, respectively, for both renewable and nonrenewable energy firms. The findings show that a 1% significance increase in liquidly will decrease ROE by 0. 0171% for non-renewable energy firms and 0. 0206% for renewable energy firms. Likewise, the findings show that a 1% significance level increase liquidly will decrease the ROA by 0. 00413% for non-renewable energy firms and 0.00403% for renewable energy firms. These findings affirm that liquidity adversely impacted the financial performance of the energy firms in China. Although liquidity position is important in the energy firms to measures company’s ability to pay off its short-term debt, the findings may be attributed to fact that more liquid assets in energy firms mean less yields. Besides, the findings from Table 5 show that firm size, employed as a control variable in this study, has a powerful influence on renewable energy firms only. This proves the importance of asset size in renewable energy firms. However, some renewable energy investments often require high upfront spending and funds. Thus the firm size plays a significant role in affecting the performance of the renewable energy industry. Additionally, the findings show that tangibility has an adverse influence on all types of energy firms, as stated in models 1 to 6, indicating that an increase in tangibility assets will negatively affect the financial performance for both ROE and ROA of the energy firms (renewable and nonrenewable energy firms). Moreover, the findings show that the firm age has a negative influence on the ROA for both renewable and nonrenewable energy firms.

We further extend our analyses and consider the influence of COVID-19 on the relationship between leverage and profitability measures. Table 6 reports the results. The findings reveal that the coefficients of leverage in all models are negative and statistically significant, while the coefficient of the interaction of leverage and COVID-19 is statistically positive (negative) in model 5 (6). These findings indicate that the negative impact of leverage on ROA is reduced during the COVID-19 period (-0.162 + 0.0571 = -0.1049) for non-renewable energy firms. However, the adverse effect of leverage on renewable energy firms is exacerbated during the pandemic.

**Table 6:** The impact of COVID-19 on the relationship among leverage and performance.

Dependent Variables	ROE			ROA		
	Model 1	Model 2	Model 3	Model 4	Model 5	Model 6
Constant	-0.196	0.381	-1.100	0.0197	0.261**	-0.400**
	(-0.53)	(0.85)	(-1.48)	(0.18)	(2.05)	(-2.03)
LEV	-0.492***	-0.516***	-0.344*	-0.154***	-0.162***	-0.109**
	(-6.04)	(-5.94)	(-1.71)	(-7.52)	(-7.95)	(-2.54)
COVID	-0.0247	-0.0392	0.0588	-0.0126	-0.0298**	0.0495***
	(-0.63)	(-0.81)	(0.99)	(-1.13)	(-2.34)	(2.77)
LEV * COVID	0.0283	0.0560	-0.122	0.0278	0.0571***	-0.0781**
	(0.35)	(0.55)	(-0.99)	(1.46)	(2.64)	(-2.37)
LIQ	-0.019***	-0.017***	-0.021**	-0.0042***	-0.0044***	-0.0046**
	(-4.52)	(-3.74)	(-2.34)	(-3.29)	(-2.67)	(-2.66)
Size	0.0329*	0.00545	0.0765*	0.00789	-0.00349	0.0284***
	(1.86)	(0.26)	(1.87)	(1.54)	(-0.61)	(2.82)
TAN	-0.277***	-0.188**	-0.52***	-0.0790***	-0.0505**	-0.162***
	(-3.35)	(-2.32)	(-2.80)	(-3.11)	(-2.05)	(-2.92)
Age	-0.0038	-0.0033	-0.0066	-0.0023***	-0.00183**	-0.0047*
	(-1.36)	(-1.28)	(-0.60)	(-3.06)	(-2.53)	(-1.77)
Observations	1787	1282	505	1787	1282	505
Group	163	112	51	163	112	51
R-Square	0.0829	0.0957	0.0970	0.123	0.147	0.164
Adj R-Square	0.0793	0.0907	0.0843	0.120	0.143	0.152
P-value	0.00	0.00	0.024	0.00	0.00	0.00

An interpretation of this is that the drop in energy prices, such as oil and gas prices, and demand for oil and gas in the second quarter of 2020 undoubtedly affected the performance of the global energy firms.

Table 7 reports the regression outputs on the effect of COVID-19 on the relationship between liquidity and performance. The results show that liquidity bear negative coefficients in all models. However, the interaction coefficients of Liquidity*COVID-19 are positive and statistically significant in models 3 and 6. These findings suggest that renewable energy firms with more liquidity during the pandemic reduce liquidity’s negative influence on energy firms’ performance in China. One possible interpretation of this finding is that firms with more liquidity during the pandemic can meet short-term obligations and avoid the cost incurred in loan due extension.

**Table 7:** The impact of COVID-19 on the relationship among liquidity and performance.

Dependent Variables	ROE			ROA		
	Model 1	Model 2	Model 3	Model 4	Model 5	Model 6
Constant	-0.195	0.384	-1.083	0.0198	0.265**	-0.392*
	(-0.53)	(0.85)	(-1.46)	(0.18)	(2.09)	(-1.99)
LEV	-0.488***	-0.507***	-0.365*	-0.150***	-0.152***	-0.120***
	(-5.96)	(-5.64)	(-1.82)	(-7.40)	(-7.52)	(-2.85)
LIQ	-0.0184***	-0.0170***	-0.0240**	-0.00411***	-0.00402**	-0.00546***
	(-4.55)	(-3.79)	(-2.36)	(-3.19)	(-2.50)	(-2.83)
COVID	-0.00940	-0.00358	-0.0295	0.00350	0.00555	-0.00249
	(-0.57)	(-0.18)	(-0.92)	(0.83)	(1.12)	(-0.29)
LIQ * COVID	0.0000776	-0.00308	0.0109*	-0.000525	-0.00257	0.00437**
	(0.02)	(-0.59)	(1.81)	(-0.41)	(-1.60)	(2.11)
Size	0.0328*	0.00502	0.0765*	0.00775	-0.00397	0.0284***
	(1.85)	(0.24)	(1.88)	(1.50)	(-0.69)	(2.83)
TAN	-0.276***	-0.185**	-0.528***	-0.0781***	-0.0475*	-0.163***
	(-3.33)	(-2.27)	(-2.78)	(-3.06)	(-1.94)	(-2.88)
Age	-0.00382	-0.00333	-0.00642	-0.00226***	-0.00182**	-0.00448*
	(-1.36)	(-1.28)	(-0.59)	(-3.06)	(-2.52)	(-1.73)
Observations	1787	1282	505	1787	1282	505
Group	163	112	51	163	112	51
R-Square	0.0829	0.0954	0.0974	0.122	0.144	0.160
Adj R-Square	0.0792	0.0905	0.0847	0.119	0.139	0.148
P-value	0.00	0.00	0.024	0.00	0.00	0.00

Overall, the present study shows that the leverage and liquidity of energy firms have a strong negative impact on the performance of non-renewable and renewable energy firms in China. Furthermore, it is the first to assess the effects of COVID-19 on the connection among leverage and liquidity on performance.

## 5. Conclusion

The exact impact of capital structure and liquidity on the firm performance in different sectors remains elusive because empirical works have yielded inconclusive outcomes. Besides, the impact of COVID-19 on the performance of energy firms is still limited. In this link, the study aims to present fresh insight to the frontiers of the empirical literature on the impact of capital structure and liquidity on financial performance in response to COVID-19 in the case of leading renewable and nonrenewable energy firms in China. To this end, the study uses the fixed effect approach to evaluate the impact of energy firms’ characteristics on their performance.

The empirical findings from the employed models showed that financial leverage is significantly adversely correlated with the ROE and ROA. Thus, the study affirmed that capital structure has a powerful influence on the financial performance of China’s renewable and nonrenewable energy firms. These findings are in line with the recent studies of [12], who affirmed that financial leverage has an adverse correlation with financial performance, and [9], who demonstrated that financial leverage negatively correlated with the financial performance of the energy firms in the USA.

This finding indicates that an increase in the debt level in global energy firms presents negative news for investors and stockholders, leading to a decline in the prices of stocks and profitability ratios. In this context, a high financial leverage ratio means a riskier investment because the firms may not be able to generate enough cash to repay their debts. However, a significant rise in financial leverage may decrease the management’s willingness to invest and expand in the market. In this way, if the percentage of debt exceeds a critical level in the firm, the increasing debt cost combines with a more significant financial distress risk component, which will negatively affect the firm performance. The study’s findings align with the pecking order theory, which assumes a negative link between debt ratio and profitability because firms prefer to use internal financing as the first for their investments before relying on debt financing. On the other hand, the models’ findings illustrated that COVID-19 is significantly adversely correlated with the ROE and ROA of non-renewable firms in China.

In contrast, the findings show that COVID-19 significantly correlates with the ROE and ROA of renewable firms in China. Thus, the study affirmed that COVID-19 positively impacts the performance of renewable energy. The current study’s findings are constant with (Fu & Shen, 2020;; Huang & Liu, 2021; Polemis & Soursou, 2020), who affirmed that COVID-19 affected negatively on some energy firms. The COVID-19 pandemic has restricted movement globally, resulting in a drop in oil demand in early April 2020. Drop in oil prices and pandemic restrictions negatively affected one main activity of the nonrenewable energy firms, eventually leading to a decline in liquidity and profitability ratios.

However, the paper’s outcomes suggest that capital structure decisions significantly affect the financial performance of energy firms in China. Besides, COVID-19 significantly affected the financial performance of energy firms. Based on the empirical findings, this work puts forward the following recommendation to policymakers, hoping to sustain the financial performance of the energy firms: First, a significant debt financing increase adversely influences Chinese energy firms’ performance. These findings suggest that policymakers must pursue moderate rather than excessive debt levels in the capital structure mix, especially in firms with high financial distress risk. Energy firms in China must consider internal funds as an alternative and significant tool to promote financial performance and avoid external finance constraints during periods of economic crisis.

Second, COVID-19 negatively (positively) affected the performance of nonrenewable (renewable) energy firms. These findings suggest that policymakers must design new policies to promote renewable energy firms. The present study employed the fixed effect model to assess the relationship among the tested variables. However, the limitation of this paper is that it does not include the whole energy due to the limitation of the data availability. Future empirical works can be undertaken by evaluating the effect of capital structure on the performance of leading energy firms pre-COVID-19 and post-COVID-19. The dynamic interrelationships between among governance and the performance of leading energy firms can also be explored in future research.
