# Correction: Capital structure and financial performance of China’s energy industry: What can we infer from COVID-19?

**DOI:** 10.1371/journal.pone.0316683

**Published:** 2024-12-27

**Authors:** Ahmed Samour, Abdullah AlGhazali, Mihaela Gadoiu, Mariana Banuta

There are errors in the author affiliations. The correct affiliations are as follows:

Ahmed Samour^1^, Abdullah AlGhazali^2^, Mihaela Gadoiu^3^, Mariana Banuta^3^

**1** Department of Accounting, Dhofar University, Salalah, Oman, **2** Department of Finance and Economics, Dhofar University, Salalah, Oman, **3** Department of Finance, Accounting and Economics, National University of Science and Technology Politehnica Bucharest, Pitesti University Center, Pitesti, Romania.
